# Time-lag in extinction dynamics in experimental populations: evidence for a genetic Allee effect?

**DOI:** 10.1111/1365-2656.12051

**Published:** 2013-02-07

**Authors:** Elodie Vercken, Flora Vincent, Ludovic Mailleret, Nicolas Ris, Elisabeth Tabone, Xavier Fauvergue, William Gurney

**Affiliations:** 1 INRA, UMR 1355 Institut Sophia Agrobiotech, Equipe ‘Biologie des Populations Introduites’06903, Sophia Antipolis, France; 2 Université de Nice Sophia Antipolis, UMR Institut Sophia, Agrobiotech, Equipe ‘Biologie des Populations Introduites’06903, Sophia Antipolis, France; 3 CNRS, UMR 7254 Institut Sophia Agrobiotech, Equipe ‘Biologie des Populations Introduites’06903, Sophia Antipolis, France; 4 INRIA, Biocore06902 Sophia Antipolis, France

**Keywords:** adaptation, extinction debt, inbreeding depression, inoculum size, negative density dependence, propagule size, Theta-Ricker model, *Trichogramma*

## Abstract

**1.** Propagule pressure, i.e. the number of individuals introduced, is thought to be a major predictor of the establishment success of introduced populations in the field. Its influence in laboratory experimental systems has however been questioned. In fact, other factors involved in long-term population persistence, like habitat size, were usually found to explain most of the dynamics of experimental populations.

**2.** To better understand the respective influence of short- and long-term factors and their potential interaction on extinction dynamics in experimental systems, we investigated the influence of propagule pressure, habitat size and genetic background on the early dynamics of laboratory-based populations of a hymenopteran parasitoid.

**3.** The amount of demographic variance differed between establishment and persistence phase and was influenced by habitat size and genetic background (geographic strain), but independent of propagule pressure. In contrast, the probability of extinction within five generations depended on the genetic background and on the interaction between propagule pressure and habitat size. Vulnerability to extinction in small size habitats was increased when populations were founded with a small number of individuals, but this effect was delayed until the third to fifth generations.

**4.** These results indicate that demographic stochasticity is influential during population establishment, but is not affected by the genetic variability of propagules. On the other hand, extinction might be influenced by a genetic Allee effect triggered by the combination of low propagule pressure and genetic drift. Finally, we documented consistent differences between genetic backgrounds in both deterministic and stochastic population dynamics patterns, with major consequences on extinction risk and ultimately population establishment.

## Introduction

The majority of introduced populations go extinct within a few generations and never become established (Williamson 1996; Seddon, Armstrong & Maloney 2007; Simberloff 2009). Rates of establishment range from 10% for invasive weeds (Williamson 1996; Booth, Murphy & Swanton 2003) up to 30% in conservation programs (Griffith *et al*. 1989; Wolf *et al*. 1996; Noël *et al*. 2011) and classical biological control operations (Hall & Ehler 1979; Stiling 1990). However, these average rates hide a strong heterogeneity between introduction events, and it appears difficult to identify a minimum set of robust predictors that would be highly predictive of establishment and invasion success (Kolar & Lodge 2001; Facon *et al*. 2006; Hayes & Barry 2008). Nevertheless, unsuitable environmental conditions (abiotic conditions, competing species, predation and diseases, absence of preys or mutualists) are likely to be responsible for many early extinctions in the context of accidental releases (Crawley 1987; Lodge 1993). In contrast, in the case of planned introductions, the environment is carefully selected to maximize establishment success, yet their failure rate remains high, because of the influence of intrinsic genetic and demographic characteristics of the introduced populations.

Both experimental and correlative evidence highlight the importance of propagule pressure in determining establishment success in nature (Kolar & Lodge 2001; Lockwood, Cassey & Blackburn 2005; Simberloff 2009). Propagule pressure refers to the total number of individuals introduced in a given area, which combines the number of introduction events (propagule number) and the number of individuals for each introduction event (propagule size or inoculum size, Drake, Baggenstos & Lodge 2005). When a single introduction is considered, there is consistent evidence for a positive correlation between propagule size and establishment probability (e.g. Memmott, Fowler & Hill 1998; Grevstad 1999; Berggren 2001; Forsyth & Duncan 2001; Ahlroth *et al*. 2003; Memmott *et al*. 2005). Detrimental demographic or genetic processes like demographic stochasticity, Allee effects, inbreeding depression and lower adaptive potential increase the vulnerability to extinction of initially small populations (Shaffer 1987; Lande 1988; Reed 2005; Willi, Van Buskirk & Hoffmann 2006; Fauvergue *et al*. 2012). In addition, propagule number is also likely to increase establishment success by decreasing stochasticity in space or time, and/or through more complex dynamic processes at the scale of metapopulations. The rescue effect, with repeated immigration events increasing the persistence probability of a population, is one such process (Brown & Kodric-Brown 1977; Hanski 1998).

In contrast with field studies of introduced populations, the analysis of extinctions in laboratory-based experimental systems indicate that habitat capacity and dynamic regime are consistent predictors of extinction probability (Philippi *et al*. 1987; Forney & Gilpin 1989; Belovsky *et al*. 1999; Desharnais *et al*. 2006), while the influence of propagule pressure is less strongly supported (Burkey 1997; Belovsky *et al*. 1999; Griffen & Drake 2008; but see Drake, Baggenstos & Lodge 2005). Several phenomena can explain the discrepancy between natural and experimental populations. First, experimental microcosms can produce abnormally high densities and therefore prevent most common types of Allee effects such as mate finding failure. Moreover, the spatial or temporal homogeneity in laboratory experiments tends to minimize inter-individual variance in reproductive success, i.e. demographic stochasticity. Finally, conditions in controlled microcosms are usually optimal (absence of predation, high quantity/quality of resources), so that the relative importance of detrimental demographic processes is likely to be attenuated.

This contrasted environmental conditions between laboratory and natural populations also affect the relative durations of the two consecutive phases of the introduction process: the ‘establishment phase’, occurring immediately after introduction and the ‘persistence phase’, occurring once the carrying capacity is reached (Drake, Shapiro & Griffen 2011). In natural populations, the establishment phase can last very long as a result of low initial population growth rate (Caley, Groves & Barker 2008). In experimental populations, initial population growth rate is close to maximal (Griffen & Drake 2008), so that the establishment phase is usually very short compared with the persistence phase, and most extinctions occur during the latter. Thus, natural environments are more prone to unveil extinction causes pertaining to the establishment phase, which are related to propagule characteristics. In contrast, factors influencing the persistence phase, as determined by habitat properties, should be more easily detected in controlled environments. As a consequence, studying populations' extinction in natural or controlled environments can be biased towards propagule or habitat related factors respectively (Drake, Shapiro & Griffen 2011).

To better understand the interplay between factors driving extinction dynamics during the establishment and the persistence phase, we investigated the determinants of establishment success in laboratory populations of a hymenopteran parasitoid during the very first generation after introduction. Experimental populations of the parasitoid were initiated with different numbers of mated females (propagule pressure) with two levels of host availability (habitat size). This design allowed us to investigate the expected differential influence of both factors on the ‘establishment’ and ‘persistence’ phases as well as possible interactions. This experiment was replicated for three different geographic strains to estimate the influence of genetic background on the relationship between propagule pressure, habitat size and extinction dynamics. Levels of genetic load (i.e. decrease in fitness in highly homozygous individuals) in the three strains were compared in parallel.

Two main population dynamics' features, demographic stochasticity and extinction probability in the first generation, were more precisely investigated with regard to theoretical expectations. We analysed whether the amount of individual demographic variance was higher during the establishment or the persistence phase, and whether it was influenced by propagule pressure or habitat size. Then, we analysed the respective influence of propagule pressure and habitat size on extinction probability. A significant effect of propagule pressure on extinction probability would imply that the extinction risk is driven mostly by the dynamics of the establishment phase. Alternatively, a significant effect of habitat size would imply that extinction risk is rather determined by the persistence phase. Interestingly, a significant interaction between propagule pressure and habitat size on either demographic stochasticity or extinction probability would indicate that population dynamics during the two phases are inter-dependent, i.e. that equilibrium dynamics are influenced by initial conditions.

## Materials and Methods

### Study system

We used the wasp *Trichogramma chilonis* (Hymenoptera: Trichogrammatidae) as a model species. *Trichogramma* are minute solitary parasitoids of Lepidopteran eggs widely used as biological control agent against noxious species (Smith 1996). The widespread species *T. chilonis* is more specifically used against the sugar cane spotted borer *Chilo sacchariphagus* (Tabone *et al*. 2010).

Three dioecious strains of *T. chilonis* were used for this experiment. Sexual reproduction in the Hymenoptera is characterized by arrhenotokous haplodiploidy, that is, fertilized eggs develop into diploid females and unfertilized eggs into haploid males (Quicke 1997; Heimpel & de Boer 2008). As a consequence, mated females have a direct control over the sex of their progeny by choosing whether they fertilize each egg or not, and highly skewed sex-ratios can sometimes be observed (Hamilton 1967; Godfray 1990). In *Trichogramma* species, sex-ratios are often biased towards females, except in the presence of superparasitism where sex-ratios tend to be skewed towards males as a result of better larval survival of males (Bonnet 2010).

The three strains were founded from individuals caught in the field in Taiwan (in 1987), Reunion Island (in the Indian ocean, in 1998) and Vietnam (in 2000, Benvenuto *et al*. 2012). The strains were then maintained in laboratory conditions on the factitious host *Ephestia kuehniella* (flour moth). Recent studies based on molecular and phenotypic characteristics have evidenced that these three strains were significantly differentiated (Benvenuto *et al*. 2012) although no morphological differences can be observed (B. Pintureau, pers. comm.). Sequences of the cytochrome *c* oxidase subunit I for the three strains obtained with the primers LCO1490 and HCO2198 (Cheyppe-Buchmann *et al*. 2011) are available on GenBank (Reunion: JQ598733-JQ598741; Taiwan: JQ598722-JQ598728; Vietnam: JQ598709-JQ598711).

For this experiment, temperature and light conditions were set on a cycle of 16 h daylight (25 °C)/8 h dark (20 °C) with constant 70% humidity. Under these conditions, generation time was 9 days for the Taiwan strain, 10 days for the Reunion strain and 12 days for the Vietnam strain.

### Experiment 1. Effect of genetic background, propagule pressure and habitat size on population dynamics

To study the influence of genetic background, propagule pressure, habitat size and their interaction on population dynamics, we set up a 3 × 5 × 2 factorial design (30 combinations). The three factors were the geographic strain (Reunion, Taiwan or Vietnam), the number of founding females (1, 2, 5, 10 or 20 mated females) and the level of host availability (low, 2 host patches; high, 10 host patches). In what follows, differences in host availability are referred to as differences in habitat size (i.e. biological habitat), while the abiotic environment such as the volume of each experimental unit is constant across treatments. Each combination of strain, propagule pressure and habitat size was replicated four times, except for the treatment level with 20 founding females, which was replicated only twice because of the limited number of individuals available for this experiment. In total, 108 populations were studied.

Each experimental population unit was contained in a plastic tube (diameter 50 mm, length 100 mm). Food for adults was provided *ad libitum* as drops of honey placed on the tube walls. Hosts were provided as 3 mm-diameter patches of *E. kuehniella* eggs stuck on paper strips (either 2 or 10 egg patches on a 10 × 50 mm paper strip; average number of eggs on a patch and 95% confidence interval: 45·8 [44·5–47·1], *n* = 65). The variation in the number of eggs across patches can be considered as a source of environmental stochasticity. However, as the range of these variations was very small (around 2%) and they were randomly distributed among experimental treatments, it is unlikely to have had any strong influence on population dynamics.

Host patches were exposed 48 h to parasitoids and then put aside until parasitoid emergence. Population size at the next generation was estimated by counting the total number of parasitized eggs before emergence. Parasitized eggs turn black when the parasitoid reaches the nymphal stage, i.e. after intra-host larval mortality has eventually occurred, so the number of black eggs directly reflects adult population size at emergence. At the beginning of emergence, fresh host patches were introduced in the tubes and similarly exposed during 48 h to obtain the next parasitoid generation.

These experimental populations are thus based on a host–parasitoid system where only parasitoid population size varies between generations following density-dependent dynamics, while host population size remains constant. Parasitoid population dynamics were recorded during the first five generations after introduction between March and May 2011. Because generation times were different for the three geographic strains, experimental populations were founded on different days during the first 2 weeks of March. Therefore, population dynamics were not exactly synchronized across strains, so that repeatable patterns of dynamics across strains did not reflect higher level variations from a shared environment.

### Analysis of demographic stochasticity

The analysis of demographic stochasticity was based on variations in population size. Because extinction events were analysed separately (see next section), we analysed variations in population size during five generations, or until the last generation before extinction. To ensure statistical independence, all replicates were divided into two different subsets, one being used for the model fitting and parameter estimation and the other to estimate independently the level of demographic stochasticity and to test hypotheses about the determinants of demographic stochasticity.

For each strain and each level of habitat size, we compared the fit of six alternative population dynamics models that describe the relationship between population size and per capita growth rate (Table 1) using the subset of data for model fitting. Model parameters were estimated using nonlinear least squares regression (procedure ‘nlminb’ in the R statistical package) and model fits were compared with lowest AIC criterion (Table S1).

**Table 1:** Models of population dynamics tested to describe experimental variations in population size

Model	Recurrence equation	Parameters	Processes described
Geometric	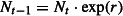	*r* exponential growth rate	Unlimited growth of the population: constant per capita growth rate
Ricker (Ricker 1954)	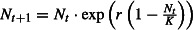	*r* exponential growth rate *K* carrying capacity	Negative density dependence: the natural logarithm of the per capita growth rate decreases linearly with population size
Theta-Ricker (Gilpin & Ayala 1973)	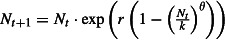	*r* exponential growth rate *K* carrying capacity *θ* coefficient of curvature	Negative density-dependence: the natural logarithm of the per capita growth rate decreases nonlinearly with population size
Classical Allee effect (Lewis & Kareiva 1993)	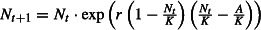	*r* exponential growth rate *K* carrying capacity *A* Allee threshold	Positive and negative density dependence: the relationship between population size and per capita growth rate is hump-shaped. If A > 0, the Allee effect is strong. If A < 0, the Allee effect is weak.
Allee-Ricker (Avilés 1999)	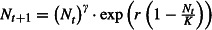	*r* exponential growth rate *K* carrying capacity *γ* cooperation parameter	Positive and negative density dependence: the relationship between population size and per capita growth rate is hump-shaped. The Allee effect is always strong.
Allee- Theta-Ricker (Avilés 1999; Courchamp, Berec & Gascoigne 2008)	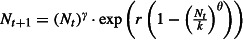	*r* exponential growth rate *K* carrying capacity *γ* cooperation parameter *θ* coefficient of curvature	Positive and negative density dependence: the relationship between population size and per capita growth rate is hump-shaped. The Allee effect is always strong.

The amount of demographic stochasticity is classically measured as the variance in the number of descendants of an individual of a population, what is called demographic variance. Following Drake (2005), we estimated this variance by comparing the best-fitting model to the subset of data used for model testing. For each experimental population *i* and at each generation *t*, we computed 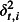, the squared residual between the observed per capita growth rate 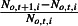 and the per capita growth rate predicted by the best fitting model initialized at the observed size of the population in generation *t*: 
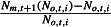
 These residuals are representative of population's variance in the per capita growth rate rather than demographic variance: they estimate the variance of the mean of the per capita growth rate over the individuals of the population. Because the variance of the mean of a random variable is equal to the variance of the random variable divided by sample size, population's variance in the per capita growth rate is equal to demographic variance scaled by the size of the population (May 1973; Lande, Engen & Saether 2003). Thus, to remove this scaling effect, we multiplied each squared residual 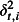 by the size of the population *N*_*o*,*t*,*i*_ to obtain the rescaled residuals 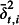 (Drake 2005).

In all combinations of geographic strain and habitat size, populations reached carrying capacity within two generations after introduction (Fig. S1). Therefore, we defined the establishment phase as the first two generations, and the persistence phase as the generations three to five. We analysed the influence of dynamical phase, in interaction with geographic strain and either propagule pressure or habitat size on the logarithm of the rescaled residuals 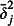. The latter followed a Gaussian distribution and was analysed with a linear model (procedure ‘lm’ in R). The complete statistical model including all interactions was compared with all sub-models using lowest AIC criterion to select the optimal combination of explanatory variables (Table S2).

### Analysis of extinction probability

Extinction was considered as a binary variable describing the fate of populations at the fifth generation (0: persisting population; 1: extinct population), and was analysed with generalized linear models. Deterministic population dynamics and random demographic stochasticity are both expected to influence strongly the probability of population extinction. Therefore, we included populations' coefficient of variation as a synthetic covariate to account for these effects.

In contrast with demographic stochasticity, which is an instantaneous process, extinction events resulting from demographic processes related to establishment can occur at later generations. Therefore, we could not analyse extinction events based on their generation of occurrence. However, as we expected extinctions to be influenced by propagule pressure during the establishment phase and habitat size during the persistence phase, we used these two variables as indicators of the different phases of extinction dynamics. We thus tested for the effect of the interaction between propagule pressure and habitat size (as well as geographic strain) on extinction probability. We selected the best model among all candidate models using lowest AIC criterion (Table S3).

### Experiment 2. Inbreeding depression

To test for the presence of different levels of inbreeding depression between strains, we founded experimental populations with a single virgin female and either (i) one of her brothers or (ii) a male selected at random from the same strain. Habitat size was set as one host patch and population dynamics were followed across three generations after introduction.

In this experiment, host eggs were sorted and isolated before parasitoid emergence to control mating. To found each population one female and one male were introduced simultaneously in a tube. Therefore, individuals were more manipulated than in the previous experiment, and females were not mated prior to population foundation, which could affect the probability that they successfully initiate a population. In particular, a female could remain unmated if the male was rejected or died before mating had occurred. In this case, the population would get extinct after one generation made of only male individuals. To account for these random events, we set aside the populations where host was parasitized (initial extinction) and those where females were not mated (i.e. only males emerged from the first generation of parasitized hosts). We excluded such populations (unsuccessful foundation). For the analysis of successfully founded populations, we examined the following components of population dynamics: (i) initial growth rate (population size at the first generation), which reflects the maximum growth rate in a non-limiting environment; (ii) probability of cumulative extinction at the third generation (probability that a population became extinct at the second or the third generation); (iii) population sex-ratio in all generations. Probabilities and sex-ratio were modelled as binomial variables, and population size as a Poisson variable. All variables were analysed with generalized linear models. In all analyses, we tested for the effect of cross (inbred or random), strain and their interaction on the response variables. Generation was added as a covariate in the analysis of sex-ratio. We selected the best model among all candidate models using lowest AIC criterion.

## Results

### Model fitting of experimental population dynamics

For all combinations of strain and habitat size, the Theta-Ricker model was selected as the best-fitting model (Table S1). This model assumes negative density dependence, which suggests that population growth is limited by intraspecific competition and that there is no Allee effect at low density. Nevertheless, because the founding females were collected already mated from rearing populations, one could argue that the first generation reflected high-density conditions and thus could not be used to detect a potential Allee effect. Therefore, we re-analysed the fit of population dynamics models when excluding the first generation, to check whether this partial data set provided stronger support for the presence of an Allee effect. This was not the case, as a simple Ricker model was selected when excluding the first generation (Table S1), thus confirming our experimental populations were mainly impacted by negative density dependence. Therefore, we chose to keep the larger data set including the first generation for the analysis of demographic stochasticity.

### Demographic stochasticity

**Fig. 1:**
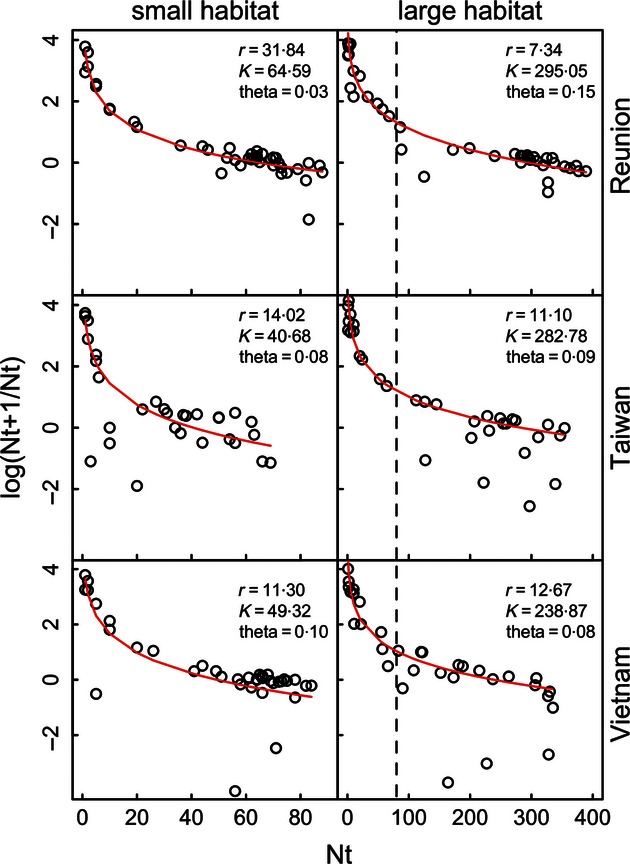
Projection of the natural logarithm of the ratio of population sizes between two consecutive generations in function of population size. Under this transformation, simple negative density dependence (Ricker model) appears as a straight, decreasing line, while nonlinear density dependence (Theta-Ricker model) is either a decreasing concave function (*θ* > 1) or a decreasing convex function (*θ* < 1). A Theta-Ricker model with *θ* < 1 was fitted for all combinations of strain and habitat size (model fit is drawn in red). The scale of the left panel is represented by a dashed line on the right panel to highlight the stronger density dependence regulation exerted in the small habitats.

**Fig. 2:**
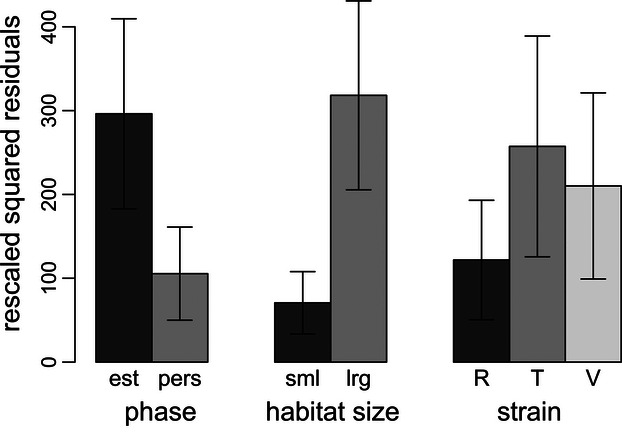
Mean rescaled squared residuals between predicted and observed dynamics along the five generations: (left) during establishment or persistence phase; (middle) in small and large habitats; and (right) in populations of the three geographic strains (R: Reunion, T: Taiwan, V: Vietnam). Error bars are 95% confidence intervals.

In our time series, the global pattern of population variation was well captured by deterministic dynamics (the adequacy between Theta-Ricker model fits and experimental data, using data set for model testing, is presented on Fig. 1), thus we considered that the residuals between the predicted and observed per capita growth rate at each generation were representative of stochastic variance. The amount of variance in per capita growth rate is classically predicted to correlate inversely with population size (May 1973; Desharnais *et al*. 2006). Once this classical scaling effect was removed, the remaining, unexplained variation (i.e. the rescaled residuals 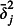) was assumed to reflect true demographic stochasticity. We found that the rescaled squared residuals depended on the dynamical phase (F_1 224_ = 17·77, *P* = 3 × 10^−5^): population trajectories were less predictable during establishment than during persistence phase (Fig. 2, left). In contrast, propagule pressure had no influence on stochastic variations (F_1 224_ = 1·17, *P* = 0·28). In addition, the rescaled squared residuals were higher in large than in small habitats (F_1 224_ = 20·19, *P* = 1 × 10^−5^, Fig. 2, middle). A possible explanation for this phenomenon is related to the intensity of density-dependent regulation (Lande, Engen & Saether 2003). In small habitats, the performance of individuals is likely to be more strongly constrained by resource competition, thus inter-individual variance in reproductive success should be lower. Finally, the demographic variance also depended strongly on geographic strain (F_2 224_ = 7·95, *P* = 4 × 10^−4^), the Reunion strain displaying on average a much lower amount of demographic stochasticity than the Taiwan and the Vietnam strain (Fig. 2, right).

### Extinction probability

On the one hand, we found that the extinction probability differed between geographic strains (χ^2^_2_ = 7·29, *P* = 2·6 × 10^−2^). Populations of the Reunion strain went extinct much less frequently (5% at the fifth generation) than populations from Taiwan or Vietnam (40–60%) (Fig. 4). This effect on extinction probability was independent of the differences in demographic stochasticity between strains, as these were encapsulated within the populations' coefficient of variation.

**Fig. 3:**
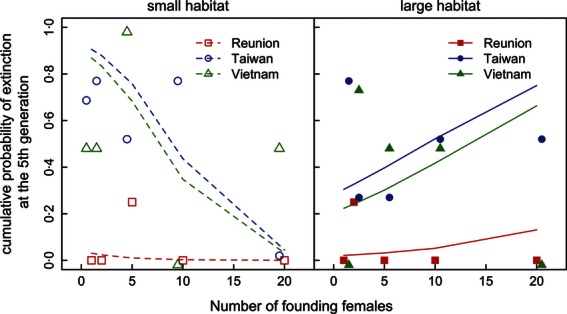
Cumulative probability of extinction at the fifth generation in small (left) and large habitats (right) in function of the number of founding females. Symbols: experimental observations averaged over all replicates. Lines: model fits including the interaction between habitat size and propagule pressure.

**Fig. 4:**
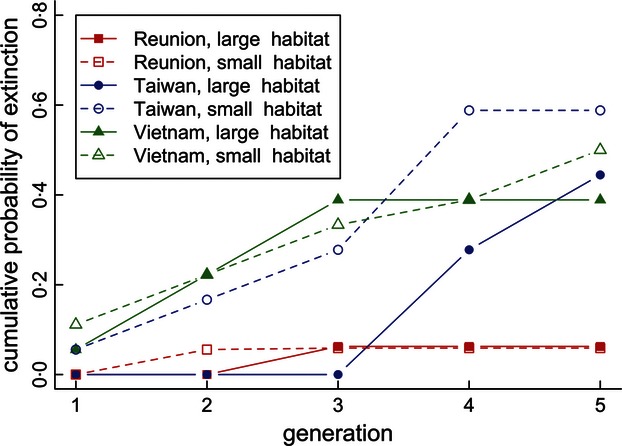
Cumulative probability of extinction along the five generations in small (dashed lines) and large habitats (full lines) for the three geographic strains.

On the other hand, we observed a significant interaction between propagule pressure and habitat size on extinction probability (χ^2^_1_ = 8·034 *P* = 4·6 × 10^−3^). In small habitats, the probability of extinction was much higher for populations founded with a low number of females (5 or less, Fig. 3 left). In contrast, the number of founding females had no influence on the probability of extinction in large habitats (Fig. 3 right). Thus, the effect of propagule pressure on establishment success was observed only in populations with small habitat size. Furthermore, the majority of extinctions occurred at the third generation or later (test for different extinction probabilities in generations 1–2, i.e. establishment phase, vs. 3–5, persistence phase: χ^2^_1_ = 4·64, *P* = 0·03, Fig. 4). These extinctions occurred after populations had reached carrying capacity (Fig. S1), which reveals a delayed effect of initial propagule pressure on extinction dynamics.

### Inbreeding depression

To understand better the differences in demographic stochasticity and extinction patterns between strains, we compared the impact of inbreeding on several components of population dynamics for the three geographic strains. The type of cross (inbred or random) had no influence on any of the variables analysed, alone or in interaction with geographic strain (all *P* > 0·25). Therefore, there is no significant inbreeding depression in any of the strains, and the different patterns in population dynamics between strains reported above are independent of their genetic load.

In this analysis, we found again significant differences between geographic strains on almost all components of population dynamics. First, the probability of initial extinction (i.e. the probability that no egg was laid at the first generation) was lower for the Taiwan strain than for the other strains (χ^2^_2_ = 7·50, *P* = 0·025), while the probability of initial female mating was the same for all strains (*P* = 0·14). Then, for populations that were successfully founded, we observed a lower initial rate of increase for the Reunion strain (χ^2^_2_ = 10·89, *P* = 4 × 10^−3^), and a higher rate of extinction for the Vietnam strain (χ^2^_2_ = 26·21, *P* = 2·0 × 10^−6^). Finally, the proportion of males increased over generations (χ^2^_1_ = 22·45, *P* = 2·14 × 10^−6^), but was lower in the Reunion strain (χ^2^_2_ = 34·26, *P* = 3·63 × 10^−8^). This analysis confirms that the three geographic strains display consistent genetic differences on several components of population dynamics, but that these differences do not result from the amount of genetic load accumulated by the strains.

## Discussion

In the first generation after introduction, extinction in our experimental populations depended on the combination of propagule pressure and habitat size. In small habitats, populations initiated with only a few females had a higher probability of extinction, while no such relationship was found in larger habitats. Demographic stochasticity can be ruled out as a major mechanism for these early extinctions, as the amount of stochastic population variation over the five generations was independent of propagule pressure. Rather, the effect of propagule pressure on extinction probability appeared after several generations and suggests the influence of a genetic factor. In addition, as both demographic stochasticity and extinction risk varied consistently between geographic strains, our results reveal a potential interaction between the demographic and genetic components of propagule pressure.

### Variation in demographic stochasticity among genetic strains

Demographic stochasticity is often pictured as a purely mechanistic process arising from limited numbers of individuals (integer arithmetic and sampling variance) and with little influence of population ecology. However, demographic stochasticity ultimately results from variance in reproductive success between individuals, which can be open to the influence of several environmental, demographic or genetic factors. For instance, we found that per capita demographic variance (i.e. rescaled squared residuals) was stronger in large habitats than in small habitats, which may reflect different competition intensity.

More interestingly, we found that variability in per capita reproduction was stronger during the establishment phase than during the persistence phase. The ecological conditions of the establishment phase, such as a novel environment or a low population density, seem to have amplified individual differences in performance. A possible explanation is that populations in the establishment phase have experienced new selective pressures, under which some individuals performed better than others as a consequence of their genetic background. Such selective sieve would have resulted in increased variance in reproductive success between individuals, i.e. increased demographic stochasticity. This hypothesis is further supported by the fact that we found that demographic variance also differed between strains, with the Reunion strain being less variable than the two other strains. Heterogeneity in demographic variance among strains of a single species has, as far as we know, never been described as such in an experimental system, and could reflect interesting differences in their adaptive history before introduction, in the field or during mass-rearing. Indeed, if the genetic variants present in the Reunion strain were on average more adapted initially to the ecological conditions of the establishment phase, individual reproductive success in the first generation should have been less variable in this strain. This is exactly what we found. Furthermore, populations from the Reunion strain also displayed the highest establishment success, with little effect from initial population size and habitat size, which could reflect pre-adaptation of this strain to the environment of introduction.

However, other non-exclusive processes can also affect the amount of inter-individual variance in reproductive success and differ between populations or strains. For instance, genetic heterogeneity is also expected to modify the amount of inter-individual variance (Kendall & Fox 2002, 2003; Robert, Sarrazin & Couvet 2003; Vindenes, Engen & Saether 2008). However, if genetic diversity *per se* were responsible for the observed differences between strains, these differences would be most apparent in populations initiated by a large number of females, where differences between strains in genetic diversity would be maximized. Indeed, in large founding populations, a low genetic diversity is expected in populations initiated from low diversity strains, while a high genetic diversity is expected in populations from the more diverse strains. In contrast, populations initiated with only one or two females should behave more similarly as their level of genetic diversity would be low as a result of the founder effect whatever the diversity of the strain of origin. This is not what we found, as the amount of demographic stochasticity was not affected by the interaction between the number of founding females and geographic strain. Alternatively, infection by bacterial endosymbionts is another potential source of variance in reproductive success among individuals. It has been demonstrated that heterogeneity in *Wolbachia* infection level associated with random maternal transmission can result in increased variance in incompatibility patterns (Guillemaud & Rousset 1997), and thus in reproductive success. Preliminary analyses failed to detect *Wolbachia* in *T. chilonis,* yet other endosymbionts like Spiroplasma, whose infection rate appears heterogeneous within and between *T. chilonis* strains (Al Khatib 2011), could be associated with the same phenomenon (Watts *et al*. 2009; Tabata *et al*. 2011).

Thus, while its underlying mechanism requires further investigation, the detection of variable demographic stochasticity between *T. chilonis* strains provides evidence for a complex relationship between population genetics, ecology and dynamics. In our system, population growth of the different strains is characterized by different values in life-history parameters like intrinsic growth rate or host saturation, which affects their deterministic dynamics pattern. In addition, the stochastic component of their population dynamics is also subject to genetic differences, which might reflect differences in pre-adaptation of the strains to their introduction environment. These two effects combined should have major consequences on extinction patterns and establishment success, and constitute a promising avenue of research for the biology of introduced populations.

### Effect of initial population size on extinction probability

It has been suggested that most laboratory experiments failed to detect an effect of initial population size on extinction probability because initially small populations achieved high population growth and quickly escaped from the demographic window where demographic stochasticity is a severe threat to population persistence (Belovsky *et al*. 1999; Griffen & Drake 2008; Drake, Shapiro & Griffen 2011). Therefore, for species like *Trichogramma* with high intrinsic rate of increase in laboratory microcosms, initial population size should influence population extinction only during the very first generation (Drake, Shapiro & Griffen 2011).

However, an apparent paradox of our results is that initial population size affected extinctions that occurred after several generations, when populations had reached carrying capacity and the initial difference in population size had been made up for. In addition, this effect of initial population size occurred only in small habitats and for two of the three strains, suggesting both environmental and genetic influences. In our data, population variability, although a major predictor of extinction probability, was independent of initial population size. Thus, the effect of propagule pressure on extinction is not mediated by demographic stochasticity. We also excluded the hypothesis of strongly deleterious recessive alleles, as populations had similar extinction dynamics, be they founded by genetically related or unrelated individuals.

Two major, non-exclusive mechanisms could have generated such a delayed response in extinction dynamics. First, deterministic population dynamics patterns in the presence of strong density dependence can be considered. In small habitats, intraspecific competition is much more intense than in large habitats (Fig. 1), and competition between females at a given generation impacts essentially larval survival, i.e. population size at the next generation. Such a negative feedback of the actual population onto its offspring associated with strong regulation can give rise to complex, divergent, population dynamics that are highly sensitive to initial conditions (May 1974). In this case, the conditions of high competition in the small habitats could have substantially amplified the small differences in propagule pressure across populations. Furthermore, we found that the amount of stochastic population variation was higher during the establishment phase. The establishment phase is thus characterized by relatively unstable, transitory dynamics, which could have interacted with density-dependent regulation to result in delayed extinction trajectories.

Alternatively, a genetic Allee effect expressed under conditions of limited population growth in the Taiwan and the Vietnam strains could also be implicated. In the smallest habitats, genetic diversity is expected to decrease quickly under the influence of genetic drift, which would bring populations initiated with a small number of individuals to a very low level of genetic diversity. The variations in propagule pressure for populations in the small habitat are thus likely to result in major differences in effective population sizes within a few generations, although the apparent population sizes might be similar (Frankham, Ballou & Briscoe 2009). In this case, the populations with the smallest effective population sizes would be most sensitive to Allee effects (Courchamp, Berec & Gascoigne 2008). This phenomenon could be described as a particular case of genetic extinction debt, which occurs whenever there is a delay between the appearance of a factor leading to population extinction and the actual extinction or decline of the population (Diamond 1972; Tilman *et al*. 1994). Under this scenario, extinctions in our experimental system would also illustrate another classical concept of conservation biology: the extinction vortex, where in our case the deleterious effects of a demographic bottleneck and low genetic diversity would be enhanced in a habitat where population growth is strongly constrained. These interesting hypotheses highlight the need for further studies to investigate the genetic mechanisms driving differences in population dynamics between different geographic strains in this species.

### Perspectives for biological control introductions

In the preliminary phases of biological control introduction programs, different species or geographic strains of natural enemies are tested and compared in the laboratory for several traits that are expected to affect introduction success and control efficiency. Resistance to cold or longevity under starvation can affect the survival of individuals during mass-rearing, storage and transportation, while fecundity, development time, mating strategies or host searching mechanisms would determine realized parasitism rate in the field. In the case of classical biological control, establishment probability can also be maximized, for instance by increasing propagule pressure (e.g. Hopper & Roush 1993; Grevstad 1999; Memmott *et al*. 2005). However, while evidence for positive effects of high propagule pressure on demographic and environmental stochasticity or Allee effects is starting to accumulate (e.g. Grevstad 1999; Memmott *et al*. 2005; Grevstad, Coombs and McEvoy in press.), the importance of genetic components (genetic diversity, genetic load, abundance and diversity of endosymbionts.) remains widely unknown (Fauvergue *et al*. 2012). In an experimental introduction of the parasitoid *Aphelinus asychis* to control the Russian wheat aphid *Diuraphis noxia*, Fauvergue & Hopper (2009) documented a persistent effect of the initial number of individuals on population growth, although populations had eventually reached the same size and all suffered from severe negative density dependence. The authors suggested that a genetic Allee effect could be responsible for their observation. Our experimental results converge to the same conclusion, and bring further support for the influence of genetic diversity on establishment success.

In addition to such effects related to genetic diversity (i.e. ‘quantitative’ genetic effects), qualitative genetic differences between individuals can also determine establishment rate, yet these have never been considered so far. In our experiment, population variability was found to be a major predictor of extinction, and differed significantly between geographic strains. The Reunion strain was characterized by a lower amount of demographic stochasticity in population dynamics; it was also less prone to extinction and its establishment success was affected by neither initial population size nor habitat size (Figs 3 and 4). This strain was thus the most efficient for experimental introduction, and was also the most resistant to demographic factors affecting establishment success. Such genetically based differences in establishment dynamics could not only be detected and selected in preliminary tests, but the performances of the best strain might even be improved through a few steps of experimental selection, to optimize further the introduction of biological control agents.

## Conclusion

The analysis of the first generations after introduction revealed a positive effect of the initial number of females on population persistence in a laboratory experimental system, which, although consistent with field introduction results, was mostly noticeable in highly competitive habitats. However, we observed a delay of three to five generations between introduction and population extinction, which suggests the existence of a genetic Allee effect in our system, alone or in combination with unstable dynamics related to strong competitive pressure. Genetic effects also determined the intensity of stochastic components in population dynamics, with major consequences on population variability and persistence. These results suggest that (1) to detect potential delayed effects, the analysis of population establishment should consider longer time-scales than the very first generations; (2) the genetic characteristics of individuals or strains should be carefully evaluated to investigate properly the interactions between demography and genetics in introduced populations. Finally, our study demonstrates that the analysis of experimental introductions can be used to investigate predictions from several conceptual frameworks, such as conservation biology, invasion biology or biological control, and to unify the theory of small, introduced populations.

## Supporting Information

**Table S1.** AIC scores for the six different population dynamics models calculated on the subset of data for model fitting (NC : the model did not converge). The Theta-Ricker model is selected for all combinations of strain and habitat size (lowest AIC indicates better fit). The same analysis was done when excluding the data for the first generation (scores in italics) : the Ricker model is selected in this case, thus confirming the absence of a typical pattern of Allee effect.

**Table S2.** Model selection for the analysis of demographic stochasticity (AIC score). The selected model appears in bold (lowest AIC scores indicates best fit).

**Table S3.** Model selection for the analysis of extinction probability (AIC score). The selected model appears in bold (lowest AIC scores indicates best fit). Population's coefficient of variation is always included as a covariate.

**Fig. S1.** Variations in experimental population sizes for all combinations of strain, habitat size and propagule pressure.

## References

[b1] Ahlroth P, Alatalo RV, Holopainen A, Kumpulainen T, Suhonen J (2003). Founder population size and number of source populations enhance colonization success in waterstriders. Oecologia.

[b2] Al Khatib F (2011). Apports des marqueurs moléculaires pour la caractérisation intra- et interspécifique des auxiliaires de lutte biologique et leurs symbiotes.

[b3] Avilés L (1999). Cooperation and non-linear dynamics: an ecological perspective on the evolution of sociality. Evolutionary Ecology Research.

[b4] Belovsky GE, Mellison C, Larson C, Van Zandt PA (1999). Experimental studies of extinction dynamics. Science.

[b5] Benvenuto C, Tabone E, Vercken E, Sorbier N, Colombel E, Warot S, Fauvergue X, Ris N (2012). Intraspecific variability in the parasitoid wasp *Trichogramma chilonis*: can we predict the outcome of hybridization?. Evolutionary Applications.

[b6] Berggren Å (2001). Colonization success in Roesel's bush-cricket *Metrioptera roeseli*: the effects of propagule size. Ecology.

[b7] Bonnet A (2010).

[b8] Booth BD, Murphy SD, Swanton CJ (2003). Weed ecology in natural and agricultural systems.

[b9] Brown JH, Kodric-Brown A (1977). Turnover rates in insular biogeography: effect of immigration on extinction. Ecology.

[b10] Burkey TV (1997). Metapopulation extinction in fragmented landscapes: using bacteria and protozoa communities as model ecosystems. American Naturalist.

[b11] Caley P, Groves RH, Barker R (2008). Estimating the invasion success of introduced plants. Diversity and Distributions.

[b13] Cheyppe-Buchmann S, Bon MC, Warot S, Jones W, Malausa T, Fauvergue X, Ris N (2011). Molecular characterization of *Psyttalia lounsburyi*, a candidate biocontrol agent of the olive fruit fly, and its *Wolbachia* symbionts as a pre-requisite for future intraspecific hybridization. BioControl.

[b14] Courchamp F, Berec L, Gascoigne J (2008). Allee effects in ecology and conservation.

[b15] Crawley MJ, Gray AJ, Crawley MJ, Edwards PJ (1987). What makes a community invasible?. Colonization, Succession and Stability.

[b16] Desharnais RA, Costantino RF, Cushing JM, Henson SM, Dennis B, King AA (2006). Experimental support of the scaling rule for demographic stochasticity. Ecology Letters.

[b17] Diamond JM (1972). Biogeographic kinetics: estimation of relaxation times for avifaunas of Southwest Pacific islands. Proceedings of the National Academy of Science of the USA.

[b18] Drake JM (2005). Density-dependent demographic variation determines extinction rate of experimental populations. PLoS Biology.

[b19] Drake JM, Baggenstos P, Lodge DM (2005). Propagule pressure and persistence in experimental populations. Biology Letters.

[b20] Drake JM, Shapiro J, Griffen BD (2011). Experimental demonstration of a two-phase population extinction hazard. Journal of the Royal Society Interface.

[b21] Facon B, Genton B, Shykoff J, Jarne P, Estoup A, David P (2006). A general eco-evolutionary framework for understanding bioinvasions. Trends in Ecology and Evolution.

[b22] Fauvergue X, Hopper KR (2009). French wasps in the New World: experimental biological control introductions reveal a demographic Allee effect. Population Ecology.

[b23] Fauvergue X, Vercken E, Malausa T, Hufbauer RA (2012). The biology of small, introduced populations, with special reference to biological control. Evolutionary Applications.

[b24] Forney KA, Gilpin ME (1989). Spatial structure and population extinction: a study with Drosophila flies. Conservation Biology.

[b25] Forsyth DM, Duncan RP (2001). Propagule size and the relative success of exotic ungulate and bird introductions to New Zealand. American Naturalist.

[b26] Frankham R, Ballou JD, Briscoe DA (2009). Introduction to conservation genetics.

[b27] Gilpin ME, Ayala FJ (1973). Global models of growth and competition. Proceedings of the National Academy of Science of the USA.

[b28] Godfray HCJ (1990). The causes and consequences of constrained sex allocation in haplodiploid animals. Journal of Evolutionary Biology.

[b29] Grevstad FS (1999). Experimental invasions using biological control introductions : the influence of release size on the chance of population establishment. Biological Invasions.

[b31] Griffen BD, Drake JM (2008). A review of extinction in experimental populations. Journal of Animal Ecology.

[b32] Griffith B, Scott JM, Carpenter JW, Reed C (1989). Translocation as a species conservation tool: status and strategy. Science.

[b33] Guillemaud T, Rousset F (1997). Consequences of Wolbachia transmission process on the infection dynamics. Journal of Evolutionary Biology.

[b34] Hall RW, Ehler LE (1979). Rate of establishment of natural enemies in classical biological control. Bulletin of the Entomological Society of America.

[b35] Hamilton WD (1967). Extraordinary sex ratios. Science.

[b36] Hanski I (1998). Metapopulation dynamics. Nature.

[b37] Hayes K, Barry S (2008). Are there any consistent predictors of invasion success?. Biological Invasions.

[b38] Heimpel GE, de Boer JG (2008). Sex determination in the Hymenoptera. Annual Review of Entomology.

[b39] Hopper KR, Roush RT (1993). Mate finding, dispersal, number released, and the success of biological control introductions. Ecological Entomology.

[b40] Kendall BE, Fox GA (2002). Variation among individuals and reduced demographic stochasticity. Conservation Biology.

[b41] Kendall BE, Fox GA (2003). Unstructured individual variation and demographic stochasticity. Conservation Biology.

[b42] Kolar CS, Lodge DM (2001). Progress in invasion biology: predicting invaders. Trends in Ecology and Evolution.

[b43] Lande R (1988). Genetics and demography in biological conservation. Science.

[b44] Lande R, Engen S, Saether B-E (2003). Stochastic Population Dynamics in Ecology and Conservation.

[b45] Lewis MA, Kareiva P (1993). Allee dynamics and the spread of invading organisms. Theoretical Population Biology.

[b46] Lockwood JL, Cassey P, Blackburn T (2005). The role of propagule pressure in explaining species invasions. Trends in Ecology and Evolution.

[b47] Lodge DM (1993). Biological invasions: lessons for ecology. Trends in Ecology and Evolution.

[b48] May RM (1973). Stability and Complexity in Model Ecosystems.

[b49] May RM (1974). Biological populations with nonoverlapping generations: stable points, stable cycles, and chaos. Science.

[b50] Memmott J, Fowler SV, Hill RL (1998). The effect of release size on the probability of establishment of biological control agents: gorse thrips (Sericothrips staphylinus) released against gorse (Ulex europaeus) in New Zealand. Biocontrol Science and Technology.

[b51] Memmott J, Craze PG, Harman HM, Syrett P, Fowler SV (2005). The effect of propagule size on the invasion of an alien insect. Journal of Animal Ecology.

[b52] Noël F, Prati D, van Kleunen M, Gygax A, Moser D, Fischer M (2011). Establishment success of 25 rare wetland species introduced into restored habitats is best predicted by ecological distance to source habitats. Biological Conservation.

[b53] Philippi TE, Carpenter MP, Case TJ, Gilpin ME (1987). Drosophila population dynamics: chaos and extinction. Ecology.

[b54] Quicke DLJ (1997). Parasitic wasps.

[b55] Reed DH (2005). Relationship between population size and fitness. Conservation Biology.

[b56] Ricker WE (1954). Stock and recruitment. Journal of the Fisheries Research Board of Canada.

[b57] Robert A, Sarrazin F, Couvet D (2003). Variation among individuals, demographic stochasticity, and extinction: response to Kendall and Fox. Conservation Biology.

[b58] Seddon PJ, Armstrong DP, Maloney RF (2007). Developing the science of Reintroduction Biology. Conservation Biology.

[b59] Shaffer M, Soulé ME (1987). Minimum viable populations: coping with uncertainty. Viable populations for conservation.

[b60] Simberloff D (2009). The role of propagule pressure in biological invasions. Annual Review of Ecology, Evolution and Systematics.

[b61] Smith SM (1996). Biological control with Trichogramma: advances, successes, and potential of their use. Annual Review of Entomology.

[b62] Stiling P (1990). Calculating the establishment rates of parasitoids in classical biological control. American Entomologist.

[b63] Tabata J, Hattori Y, Sakamoto H, Yukuhiro F, Fujii T, Kugimiya S, Mochizuki A, Ishikawa Y, Kageyama D (2011). Male killing and incomplete inheritance of a novel spiroplasma in the moth Ostrinia zaguliaevi. Microbial Ecology.

[b64] Tabone E, Bardon C, Desneux N, Wajnberg E (2010). Parasitism of different Trichogramma species and strains on Plutella xylostella L. on greenhouse cauliflower. Journal of Pest Science.

[b65] Tilman D, May RM, Lehman CL, Nowak MA (1994). Habitat destruction and the extinction debt. Nature.

[b66] Vindenes Y, Engen S, Saether B-E (2008). Individual heterogeneity in vital parameters and demographic stochasticity. American Naturalist.

[b67] Watts T, Haselkorn TS, Moran NA, Markow TA (2009). Variable incidence of Spiroplasma infections in natural populations of Drosophila species. PLoS ONE.

[b68] Willi Y, Van Buskirk J, Hoffmann AA (2006). Limits to the adaptive potential of small populations. Annual Review of Ecology, Evolution and Systematics.

[b69] Williamson MH (1996). Biological invasions.

[b70] Wolf CM, Griffith B, Reed C, Temple SA (1996). Avian and mammalian translocations: update and reanalysis of 1987 survey data. Conservation Biology.

