# Supplementary material for: Time-lag in extinction dynamics in experimental populations: evidence for a genetic Allee effect?
Source: J Anim Ecol. 2013 Feb 7;82(3):621–31. doi: 10.1111/1365-2656.12051 (PMC3708108; doi:10.1111/1365-2656.12051)
Supplement: Supplementary file 1 [file jane0082-0621-SD1.docx]

Table S1 : AIC scores for the six different population dynamics models calculated on the subset of data for model fitting (NC : the model did not converge). The Theta-Ricker model is selected for all combinations of strain and habitat size (lowest AIC indicates better fit). The same analysis was done when excluding the data for the first generation (scores in italics) : the Ricker model is selected in this case, thus confirming the absence of a typical pattern of Allee effect.

| Strain | Habitat size (# host patches) | Exponential | Ricker | Theta-Ricker | Classical Allee effect | Allee-Ricker | Allee-Theta-Ricker |
| --- | --- | --- | --- | --- | --- | --- | --- |
| Reunion | 2 | 288.8  *(210.4)* | 272.8  *(201.8)* | **248.9**  *(NC)* | NC  *(NC)* | 274.8  *(203.8)* | 250.9  *(NC)* |
|  | 10 | 421.5  *(329.3)* | 393.9  *(304.3)* | **373.1**  *(NC)* | NC  *(323.8)* | 395.9  *(306.3)* | 375.1  *(NC)* |
| Taiwan | 2 | 190.2  *(127.5)* | 174.4  *(111.1)* | **170.1**  *(112.4)* | NC  *(NC)* | 176.4  *(113.1)* | 172.1  *(114.4)* |
|  | 10 | 336.3  *(245.5)* | 321.7  *(232.5)* | **301.8**  *(NC)* | NC  *(NC)* | 323.7  *(234.5)* | 303.8  *(NC)* |
| Vietnam | 2 | 184.8  *(128.0)* | 176.7  *(126.2)* | **170.5**  *(NC)* | NC  *(127.4)* | 178.7  *(128.2)* | 172.5  *(NC)* |
|  | 10 | 299.6  *(207.7)* | 296.0  *(205.5)* | **279.1**  *(NC)* | NC  *(NC)* | 298.0  *(207.5)* | 281.1  *(NC)* |
