# Supplementary material for: Time-lag in extinction dynamics in experimental populations: evidence for a genetic Allee effect?
Source: J Anim Ecol. 2013 Feb 7;82(3):621–31. doi: 10.1111/1365-2656.12051 (PMC3708108; doi:10.1111/1365-2656.12051)
Supplement: Supplementary file 2 [file jane0082-0621-SD2.docx]

Table S2 : Model selection for the analysis of demographic stochasticity (AIC score). The selected model appears in bold (lowest AIC scores indicates best fit), and explains 20% of the total variance in the data.

| Model | AIC |
| --- | --- |
| Phase * Strain * (Habitat size + Propagule pressure) | 1122.4 |
| Phase * Strain * Propagule pressure + Phase * Habitat size | 1118.6 |
| Phase * Strain * Habitat size + Phase * Propagule pressure | 1116.3 |
| Phase * (Habitat size + Propagule pressure) + Strain | 1112.9 |
| Phase * Strain * Propagule pressure + Strain * Habitat size | 1119.7 |
| Phase * Strain * Habitat size + Strain * Propagule pressure | 1118.0 |
| Strain * (Habitat size + Propagule pressure) + Phase | 1115.6 |
| Phase * Strain * Propagule pressure + Habitat size | 1116.7 |
| Phase * Propagule pressure + Strain + Habitat size | 1112.9 |
| Phase * Strain * Habitat size + Propagule pressure | 1114.8 |
| Phase * Habitat size + Strain + Propagule pressure | 1113.3 |
| Phase * Strain + Habitat size + Propagule pressure | 1111.1 |
| Phase + Strain + Habitat size + Propagule pressure | 1111.4 |
| Strain + Habitat size + Propagule pressure | 1124.9 |
| Phase + Habitat size + Propagule pressure | 1121.1 |
| Phase + Strain + Propagule pressure | 1127.2 |
| **Phase + Strain + Habitat size** | **1108.6** |
| Phase + Strain | 1126.5 |
| Phase + Habitat size | 1120.3 |
| Phase + Propagule pressure | 1138.0 |
| Strain + Habitat size | 1124.4 |
| Strain + Propagule pressure | 1141.6 |
| Habitat size + Propagule pressure | 1136.5 |
| Phase | 1137.2 |
| Strain | 1141.1 |
| Habitat size | 1135.9 |
| Propagule pressure | 1152.3 |
| Null model | 1151 .7 |
