# Supplementary material for: Time-lag in extinction dynamics in experimental populations: evidence for a genetic Allee effect?
Source: J Anim Ecol. 2013 Feb 7;82(3):621–31. doi: 10.1111/1365-2656.12051 (PMC3708108; doi:10.1111/1365-2656.12051)
Supplement: Supplementary file 3 [file jane0082-0621-SD3.docx]

Table S3 : Model selection for the analysis of extinction probability (AIC score). Population’s coefficient of variation is always included as a covariate. The selected model appears in bold (lowest AIC scores indicates best fit), and explains 60% of the total variance in the data. Including the interactions term between propagule pressure and habitat size increases by itself the percentage of variance explained by 10% when compared with the model including additive effects only.

| Model | AIC |
| --- | --- |
| Propagule pressure * Habitat size * Strain | 76.8 |
| **Propagule pressure * Habitat size + Strain** | **68.2** |
| Propagule pressure + Habitat size*Strain | 75 .1 |
| Propagule pressure * Strain + Habitat size | 77.5 |
| Propagule pressure + Strain + Habitat size | 73.6 |
| Strain + Habitat size | 75.2 |
| Propagule pressure + Habitat size | 80.6 |
| Propagule pressure + Strain | 72.4 |
| Propagule pressure | 80.15 |
| Strain | 73.9 |
| Habitat size | 80.3 |
| Null model | 79.5 |
